# Quality of life in patients with Parkinson’s disease: Translation and psychometric evaluation of the Iranian version of PDQ-39

**Published:** 2010

**Authors:** Marzieh Nojomi, Zahra Mostafavian, Gholam Ali Shahidi, Crispin Jenkinson

**Affiliations:** a Community Medicine Specialist, Member of Mental Health Research Center, Associate Professor, Department of Community Medicine, School of Medicine, Iran University of Medical Sciences, Tehran, Iran; b Community Medicine Specialist, Iran University of Medical Sciences, Tehran, Iran; c Neurologist, Rasoule Akram Hospital, Iran University of Medical Sciences, Tehran, Iran; d Professor of Health Services Research, Health Services Research Unit, Department of Public Health, University of Oxford, Headington, Oxford, UK

**Keywords:** Parkinson’s Disease, Quality of Life, Patient Reported Outcomes Measures, PDQ-39, Validity, Reliability

## Abstract

**BACKGROUND:**

Health related quality of life is an important outcome measure in studies involving patients with chronic neurological conditions. Disease specific patient reported outcome measures (PROMs) are increasingly used as primary end points in clinical trials. The most widely used disease specific PROM is the 39 item Parkinson’s Disease Questionnaire (PDQ-39). The aim of this study was to determine validity and reliability of Persian PDQ-39.

**METHODS:**

Two hundred Parkinson’s disease patients attending neurologic clinics of teaching hospitals were recruited. PD patients completed a translated version of the PDQ-39. Internal consistency reliability of the questionnaire was assessed by Cronbach’s alpha coefficient. Reproducibility was assessed across the 3-week interval using the intraclass correlation coefficient. To assess convergent validity, results on the PDQ-39 were correlated with those gained on the SF-36. Discriminate validity of questionnaire was assessed by comparing PDQ-39 scores and the severity and the duration of disease.

**RESULTS:**

A value of 0.93 (Cronbach’s α) was gained for the summary score (PDQ-SI), indicating high levels of internal reliability. Alpha value of seven domains was greater than 0.70. The intraclass correlation coefficient ranged from 0.47 to 0.90. The range of correlation coefficients between domains of SF-36 and PDQ-SI was from -0.40 to -0.61. There was a statistically significant difference between severity of disease and mean scores of PDSI.

**CONCLUSIONS:**

This study provides evidence that the Persian version of PDQ-39 is a valid and reliable measure of quality of life in PD.

Parkinson’s disease (PD) is a chronic neurodegenerative disease. It is more common in males than females, and prevalence increases markedly with age, being extremely rare in those under the age of 45 years and being present in about 1% of those over 65 years, and 2% over 85.^1^ There is a marked apparent geographical variation, with low prevalence rates in China and Africa. This disease impacts many areas including mobility, cognitions, social function, psychological status, speech, communication and economic/ occupational functioning.^2^–^5^

The main focus in evaluation and treatment of patients with Parkinson’s disease has been on the motor disability of the disease. However, non-motor symptoms like mood changes, cognitive impairment and sleep disturbances often complicate the course of the disease and impact on the patient’s life.^6^–^8^ The aim of treatment and medical interventions is largely focused at decreasing the impact of this disease on the quality of life of affected patients. Health related quality of life (HRQoL) is considered to be an important outcome measure in studies involving patients with chronic diseases. In recent years emphasis has been placed on measuring the subjective experience of patients, incorporating the effect of the disease and its treatment from the patient’s point of view.^5^

The Parkinson’s disease Questionnaire (PDQ-39) is the most commonly used HRQoL-specific PD measure. This instrument was originally developed in the United Kingdom.^9^ The PDQ-39 was designed to be a valid and feasible questionnaire for self-completion that addresses the perceptions and concerns of individuals with Parkinson’s disease. Its primary use is intended to be in clinical trials and related forms of evaluation of interventions. This instrument has 39 items covering eight domains of quality of life. To date, the PDQ-39 has been adapted into more than 60 different cultures and languages.^10^

With the expected increase in life expectancy in developing countries, included Iran, in the next decades, the number and proportion of the elderly will also increase. Therefore, it is expected that the prevalence of chronic diseases, such as Parkinson’s disease will increase too. In order to evaluate the effectiveness of therapeutic intervention, valid and reliable patient reported outcome measures will be required.^9^

The aim of this study was to validate PDQ-39 questionnaire in Persian language by evaluating internal consistency, test/retest reliability, construct validity and discriminate validity, in a specific group of patients with a diagnosis of Parkinson’s disease attending neurology clinics.

## Methods

### 

#### Subjects

The study was conducted in outpatient clinics of the Iran University of Medical Sciences located in Tehran. The study was conducted between June 2008 and April 2009. At each center, the PDQ-39 questionnaire was administered to consecutive patients attending neurological hospital clinics with a diagnosis of Parkinson’s disease confirmed by a specialist neurologist. Informed consent was obtained from patients after aims of the study were explained to them. Patients self completed questionnaires at waiting rooms under the observation of a trained interviewer. Two hundred and forty patients were recruited into the study, and two hundred completed questionnaires (83%) were collected. Three weeks later the questionnaire was offered to a randomly selected sub sample of 15% of the original subjects in order to assess reproducibility. All of these subjects returned the questionnaires. The inclusion criteria were being less than 90 years old and having a confirmed diagnosis of Parkinson’s disease. Exclusion criteria included being illiterate, showing evidence of another major medical illness, showing cognitive impairment or any severe mentally or physically illness and having dementia. Approval for the study was granted by the institutional review board of Medical School of Iran University of Medical Sciences.

#### Measures

Two questionnaires were used. Quality of life of patients with Parkinson’s disease was assessed using the PDQ-39 questionnaire.^11^ The PDQ-39 contains eight domains: mobility (10 items), activities of daily living (6 items), emotional well-being (6 items), stigma (4 items), social support (3 items), cognitions (4 items), communication (3 items), and bodily discomfort (3 items). Previous research has suggested the questionnaire has good internal and test-retest reliability, as well as a good construct and face validity across a wide variety of languages and cultures.^9^,^12^ In this questionnaire each item scored from zero to four as the best to worst. Each domain score ranges from zero (the best QoL) to 100 (the worst QoL).

The second questionnaire was the Short-Form 36 (SF-36) health status survey, which has previously been validated in Persian.^13^ The SF-36 contains eight subscales measuring aspects of physical and mental health. Each dimension is reported on a scale of 0 to 100 with higher score reflected the better quality of life. Other variables included demographic variables (age, marital status, education, and occupation). Clinical variables, including disease duration, family history of disease and severity of disease were also collected. Disease severity was classified according to Hoehn and Yahr classification (HY).^14^ This classification has 5 stages. Stages were categorized as follow: stages I-II as mild, stage III as moderate, and stages IV-V as severe. Disease duration was categorized as ≤ 5 years and > 5 years.

#### Translation

Written permission was obtained from the original developers to proceed with the translation. The PDQ-39 was translated into Persian in parallel by two independent, native Iranian health professional translators, fluent in both English and Persian. Subsequently, two translators compared the Persian version and original English version of questionnaire. Another English speaker, with English as her first foreign language, who did not have any knowledge of the original instrument, then back translated the questionnaire into English. The backward translation was sent to the developer of the original questionnaire for comparison and his suggestions were incorporated into the final Persian version. A consensus meeting was held between the translators and investigators, during which the back-translation and the original scale were compared and any differences discussed and resolved. Pre-testing was completed with 20 patients to evaluate the comprehension and readability of the PDQ-39 Persian version. Patients were asked whether they encountered any difficulty in understanding each of the items. Patients indicated they had no problems with the measure and understood the items.

#### Statistical Analysis

Internal consistency and reliability of dimensions of the questionnaire were assessed using the Cronbach’s alpha coefficient. Alpha values equal to or greater than 0.70 were considered satisfactory. Item-total correlations over 0.70 were considered to show acceptable correlation between the items of questionnaire and the domain total to which they contribute. Reproducibility (test/retest reliability) was assessed across a 3-week interval on 30 (15%) patients using the intraclass correlation coefficient. The recommended level for group comparisons is 0.7.^15^ Convergent validity was evaluated by comparing the PDQ-39 and its subscales to related measures on the SF-36. To assess convergent validity, Pearson’s correlation coefficients were used. Due to inverse scoring between the two questionnaires, the expectation was for correlation coefficient to be negative. Discriminate validity of questionnaire was assessed by comparing PDQ-39 scores and the severity and the duration of disease. Differences between groups of severity were assessed with analysis of variance (ANOVA). Scheffè was used as a post hoc test. It was hypothesized that higher scores on PDQ-39 would be related to greater severity of disease. T-tests were used to compare scores between two groups of differing disease duration. Significance was set at 0.05 for all analyses.

## Results

Two hundred and forty people with Parkinson’s disease agreed to participate in the study. Two hundred (83%) participants completed and returned the questionnaires. Mean age was 57.3 (SD = 10.5) years (range: 27-83). The mean number of years since diagnosis was 6.5 (SD = 4.2) years. Mean age at onset of disease was 50.6 (SD = 11.0) years. Other demographic characteristics of the patients are displayed in table 1. One hundred and thirty five (67.5%) of the respondents were male. One hundred and seventy eight (89%) of patients were married. Distribution of patients according to severity by HY staging was as follow: 46.5% mild; 40% moderate; and 11.5% sever. Fifty six (28%) respondents had graduated from high school, but the majority had ceased education before then. Sixty four (32%) of the respondents were retired. Twenty one patients (10.5%) had a positive family history of PD between first degree relatives.

**Table 1:** Demographic characteristics of Parkinson’s disease patients (n = 200)

Variable	Number	%
Gender		
Male	135	67.5
Female	65	32.5
Education		
Less than high school	80	40.0
High school graduated	56	28.0
Academic	42	21.0
Unknown	22	11.0
Occupation		
Housewife	54	27.0
Retired	64	32.0
Still working	60	30.0
Unemployed	22	11.0
Marital status		
Married	178	89.0
Single	5	2.5
Other	17	8.5
Severity of disease		
Mild	93	46.5
Moderate	80	40.0
Severe	23	11.5
Unknown	4	2.0

Descriptive statistics for the PDQ-39 are reported in table 2. Data completion was satisfactory. Only one dimension had 6% missing data (mobility). Missing data for other domains was less than 5%.

**Table 2:** Descriptive statistics and reliability of PDQ-39 scales

PDQ-39 scale	Mean (SD)	95% CI	Cronbach’s a	Item-test correlation	Intraclass correlation coefficient (ICC)
Mobility	44.5 (23.1)	41.0-47.6	0.91	0.79	0.90
ADL*	39.1 (22.3)	35.2-42.0	0.88	0.83	0.47
Emotions	37.2 (21.0)	32.8-39.1	0.86	0.84	0.78
Stigma	33.9 (25.1)	29.8-37.4	0.87	0.73	0.82
Social	22.9 (20.4)	20.0-26.0	0.70	0.78	0.77
Cognitions	28.8 (16.8)	27.0-32.2	0.60	0.79	0.84
Communication	28.1 (21.0)	25.1-31.5	0.78	0.79	0.74
Body pain	42.0 (23.5)	38.5-45.9	0.75	0.76	0.75
PDSI**	35.1 (15.4)	23.1-36.7	0.93	----	0.80

* Activities of daily living

** Parkinson’s disease summary index

Internal consistency was assessed using Cronbach’s alpha and a value of 0.93 was gained for the summary score, indicating high levels of internal reliability. Alpha values for each subscale ranged from 0.91 (maximum) for mobility to 0.60 for cognitions (minimum). The intraclass correlation coefficient (ICC) assessing reproducibility at 3 weeks ranged from 0.90 for mobility to 0.47 for activities of daily living. The ICC for PDQ-SI was 0.80. Item-test correlation for all items and domains ranged from 0.73 to 0.84 (Table 2).

Subscales of the PDQ-39 and related measures of SF-36 showed a significant correlation between mobility and physical functioning of SF-36 (-0.61, p < 0.01). Also, there were a significant correlation between emotions and emotional well being of SF-36 (-0.55, p < 0.01) and body pain of PDQ-39 with pain domain of SF-36 (-0.60, p < 0.01).

Discriminate validity of PDQ-39 was assessed by comparing scores of domains and PDQ-SI by severity (Table 3) and duration of disease (data is not shown). The patients with mild, moderate, and severe stages of Parkinson’s disease had an average summary index score of 28 (SD = 14.3), 40 (SD = 12.4) and 46.7 (SD = 16.4), respectively. There was a statistically significant difference between scores of patients with mild versus moderate and severe disease (p = 0.005). The difference in score for domain of mobility was also significant between mild versus moderate and severe disease (p < 0.05). However, the difference of score between mild versus moderate for the emotions domain was not significant. No other significant differences were found between scores of other domains for moderate versus severe disease. Differences of scores for mild versus moderate and severe stages for these domains were significant (p < 0.05).

**Table 3:** Mean (SD) PDQ-39 scores by severity of disease (HY)

Domain	Mild (n = 86)	Moderate (n = 78)	Severe (n = 23)	P value
Mobility	31.7 (19.2)	53.1 (20.7)	65.5 (16.4)	0.0005
ADL*	28.3 (18.9)	46.8 (20.0)	55.9 (22.8)	0.0005
Emotions	32.2 (19.2)	38.7 (19.8)	53.2 (23.8)	0.0005
Stigma	28.3 (23.1)	39.3 (24.6)	42.3 (28.2)	0.004
Social support	18.8 (17.9)	25.2 (19.5)	34.4 (27.7)	0.005
Cognition	23.1 (14.9)	32.8 (16.3)	37.5 (19.4)	0.005
Communication	22.4 (19.0)	34.0 (21.0)	34.9 (22.2)	0.005
Body pain	34.9 (23.1)	47.9 (20.3)	50.0 (28.2)	0.005
PDSI**	28.0 (14.3)	40.0 (12.4)	46.7 (16.4)	0.005

* Activities of daily living

** Parkinson’s disease summary index

Respondents with disease duration of more than five years had lower scores on the PDQ-SI and all domains of PDQ-39 as compared to those with less than five years disease duration.

## Discussion

The present study assessed the validity and reliability of the Persian version of the PDQ-39 in 200 patients with Parkinson’s disease. This validation was performed in hospital-based clinics setting. The results from this investigation provide evidence that the translated version of PDQ-39 is a valid and reliable measure of quality of life in Parkinson’s disease patients. The internal consistency of questionnaire for the summary score was high (the Cronbabach’s α coefficient = 0.93). The internal consistency of 7 of the 8 scales was satisfactory (≥ 0.70). The internal consistency of “cognitions” was less than the level of considered satisfactory (α = 0.60). In the original validation study a value of 0.84 was gained for the summary score.^11^ The alpha coefficient for “cognitions” in the original questionnaire was 0.74.^9^ In a number of studies, the coefficient for this domain has ranged between 0.71 and 0.82.^9^,^16^,^17^ Some evidence has cast doubt on the reliability of the cognitions and social support domains with alpha values of less than 0.70 being reported.^16^,^18^,^19^

The intraclass correlation coefficient for summary score and domains of PDQ-39 ranged from 0.47 to 0.90 indicating good reproducibility of questionnaire. All domains but one gained ICCs greater than 0.70. The present study provided evidence of a strong correlation between scores on all items and their parent domains (0.73-0.84).

The PDQ-39 was compared with SF-36 in order to assess construct validity. The correlation between PDQ domains and related measures on the SF-36 ranged from -0.55 to -0.61 (the correlation is negative due to inverse scoring algorithms). These results are in agreement with those previously reported in the literature.^16^

In order to determine the discriminate validity of PDQ-39, the mean PDQ-SI was compared between categories of disease duration and HY stages. This study revealed that severity of disease affects quality of life of patients. The patients with mild stage of disease had the lower score of PDQ-SI and all domains of PDQ-39 as opposed to moderate and severe stages. This finding was similar to other studies.^16^,^20^ For 5 of 8 domains, any significant difference couldn’t be found between moderate and severe stages of disease. One explanation for this may be the similar level of quality of life of patients at the end stages of disease. Alternatively, the finding may be due to the small sample size of patients in these two stages, especially for severe stage. However, except for the emotions dimension, the Persian version of PDQ-39 did discriminate between mild versus moderate and severe stages of disease. In the study of Souza et al the impact of disease on quality of life was higher in the lower stages of disease for dimensions of emotional and cognitive aspects.^20^ They explained this finding due to emotional problems of disease at the first stages of disease. Some studies showed that the dimensions of social support and stigma are almost constant during the disease progression.^21^

The questionnaire also revealed a good discriminate ability according to the duration of disease, with patients with longer duration of disease having higher scores on the PDQ-SI (worse quality of life). This finding has been reported elsewhere.^20^

## Conclusions

In conclusion, present results demonstrate that the Persian version of PDQ-39 is useful for assessing quality of life of Parkinson’s disease patients in a clinical setting. It is a short and easily understood instrument that can be self-administered. The evidence presented here indicates that the PDQ-39 is a valid and reliable instrument that can be used in future studies of PD in Iran. This study did not assess the responsiveness of the PDQ-39. Consequently, future research should determine minimally important differences for domains of the Persian version of the questionnaire.
